# Anticancer Potential, Phenolic Profile, and Antioxidant Properties of *Synsepalum dulcificum* (Miracle Berry) in Colorectal Tumor Cell Lines

**DOI:** 10.3390/antiox14040381

**Published:** 2025-03-24

**Authors:** Josefa Quiroz-Troncoso, Nicolás Alegría-Aravena, Blanca Sáenz de Mierae, Marta Sánchez-Díez, Raquel González-Martos, Clara E. Gavira-O’Neill, Emilio J. González, Maria González-Miquel, Cristian Valdés Vergara, Gloria González-Silva, Loan Bensadon-Naeder, Javier Galeano, Carmen Ramírez-Castillejo

**Affiliations:** 1 HST Group, Centro de Tecnología Biomédica (CTB), Departamento Biotecnología-B.V. ETSIAAB, Universidad Politécnica de Madrid, 28223 Madrid, Spain; marta.sanchez@ctb.upm.es (M.S.-D.); raquel.gonzalez@ctb.upm.es (R.G.-M.); clara.gavira@ctb.upm.es (C.E.G.-O.); 2 Departamento de Oncología, Instituto de Investigación Sanitaria San Carlos (IdISSC), 28040 Madrid, Spain; nicolas.alegria.aravena@gmail.com; 3 Grupo de Biología y Producción de Cérvidos, Instituto de Desarrollo Regional, Universidad de Castilla La Mancha, 02006 Albacete, Spain; 4 Asociación Española Contra el Cáncer (AECC)—Fundación Científica AECC, 28045 Madrid, Spain; 5 Departamento de Ingeniería Química Industrial y del Medio Ambiente, ETSI Industriales, Universidad Politécnica de Madrid, 28006 Madrid, Spain; blanca.saenz@upm.es (B.S.d.M.); ej.gonzalez@upm.es (E.J.G.); maria.gonzalezmiquel@upm.es (M.G.-M.); 6 Instituto de Investigación de Enfermedades Raras (IIER-AGH), Instituto de Salud Carlos III, 28220 Madrid, Spain; 7 Centro de Investigación de Estudios Avanzados del Maule, Universidad Católica de Chile, Talca 3460000, Chile; cvaldesv@ucm.cl; 8 Laboratorio de Microbiología Aplicada, Escuela de Ingeniería en Biotecnología, Centro de Biotecnología de los Recursos Naturales (CenBio), Facultad de Ciencias Agrarias y Forestales, Universidad Católica del Maule, Talca 3466706, Chile; ggonzalez@ucm.cl; 9 Medicinal Gardens S.L. (Baïa Food), 28008 Madrid, Spain; loan@baiafood.com; 10 Grupo de Sistemas Complejos, Departamento de Ingeniería Agroforestal, ETSIAAB, Universidad Politécnica de Madrid, 28040 Madrid, Spain; javier.galeano@upm.es

**Keywords:** *Synsepalum dulcificum*, antioxidant activity, anticancer activity, colorectal cancer, apoptotic pathways, cell cycle

## Abstract

Polyphenols, recognized for their antioxidant capacity, have shown potential in improving the response treatment of various diseases, including cancer. In this context, polyphenols have the ability to induce cytotoxicity in tumor cells, making them possible complementary agents to current treatments. The present study aims to evaluate the effect of the aqueous extract of *Synsepalum dulcificum*, using the commercial product DMB^®^, on the proliferation of colorectal tumor cells. An aqueous extract of DMB^®^ was obtained, and 12 compounds were identified through high-performance liquid chromatography (HPLC), with protocatechuic acid, gallic acid, and catechin being the most prominent. Regarding cytotoxicity, the extracts reduced cell viability in the DLD-1, HT29, SW480, and SW620 cell lines, with IC_50_ values of 7, 11, 13, and 15 mg/mL, respectively. The combination of oxaliplatin with the DMB^®^ extract reduced the resistant population by up to 50% in the DLD-1 and SW620 cell lines, affecting the G_2_/M and S phases of the cell cycle, respectively. Additionally, treatment with the DMB^®^ extract induced an increase in the expression of *BCL2*, *CASP3*, and *CASP9*, suggesting a mechanism of action associated with apoptosis. The aqueous extract of *Synsepalum dulcificum* (DMB^®^) exhibited cytotoxicity in colorectal cancer cells, enhancing the effect of oxaliplatin and activating apoptotic pathways, suggesting its potential as an adjuvant in anticancer therapies.

## 1. Introduction

Colorectal cancer is one of the most common malignant cancers. It is the type of cancer with the third highest global incidence and the second highest mortality in the world [1]. Although the primary tumor is usually responsive to treatment (surgery and/or chemotherapy, radiotherapy), the development of treatment resistance and disease recurrence pose significant challenges. Tumor-initiating cells (TICs) play a crucial role in this process, as they are primarily responsible for relapses and resistance to conventional treatments. These cells have the capacity to regenerate tumors after treatment, emphasizing their importance as a therapeutic target in the fight against cancer recurrence [2].

In many cases, the side effects in patients are so harmful that it is necessary to reduce chemotherapy treatment, jeopardizing the treatments’ effectiveness. A reduction in oxaliplatin dose intensity in the palliative treatment of patients with advanced metastatic colon cancer (MCC) has been shown to negatively affect both disease progression and overall survival among these patients [3]. Therefore, it is crucial to identify new treatments or synergistic therapies that specifically target cellular resistance, allowing for a reduction in chemotherapy doses, particularly oxaliplatin, without compromising treatment efficacy.

This dose reduction of conventional oxaliplatin treatments has many future applications; for example, in patient populations ≥75 years, oxaliplatin-based regimens as first-line chemotherapy can be safely and effectively tailored by applying a reduction of anticancer drugs based on adverse events [4]. On the other hand, the effect of immunotherapy could be enhanced by promoting the infiltration of CD8+ T cells into tumors and delaying tumor progression, as has been reported in current combined chemotherapy and immunotherapy treatments. The currently used full dose of oxaliplatin induces severe immunosuppression and impairs the efficacy of the combined therapy [5].

In this context, several medications are already claiming to increase cell sensitivity, which allows for the reduction of oxaliplatin doses. For example, the drug orlistat [6] exhibits potent antitumor effects in several malignancies and could sensitize colorectal cancer (CRC) cells to oxaliplatin and induce marked synergistic apoptosis in vitro and in vivo at low doses. Another growing field is the use of natural extracts as potential adjuvants for cancer treatments. These natural extracts have been shown to have multiple bioactive properties, such as antioxidant, anti-inflammatory, and selective cytotoxic effects against tumor cells [7]. In this context, phenolic compounds and other secondary metabolites present in various plant species are being studied for their ability to enhance the efficacy of conventional treatments while reducing associated side effects [8]. A promising example of such extracts is that obtained from *Synsepalum dulcificum* (*S. dulcificum*), a plant native to west Africa known for its ability to transform sour flavors into sweet ones [9] but which also contains compounds with high therapeutic potential, particularly in cancer treatment. Its antioxidant properties, along with its potential role in sensitizing tumor cells to chemotherapy [10], make this plant an interesting candidate for future research in colorectal cancer treatment.

Initial interest in *S. dulcificum* was related to its capacity to transform the perception of acidic flavors into sweet ones due to the miraculin protein, first identified in 1968 [9]. However, the plant also contains a number of phenolic compounds with high antioxidant capacity. In contrast to other species that have undergone more exhaustive characterization, extraction of these compounds from the pulp of the fruit has not been optimized for its industrial use. In addition, its cytotoxic capacity has not been extensively characterized, although some scientific studies have demonstrated that the extract from *S. dulcificum* berries shows promising effects against colorectal cancer. These effects appear to be mediated by apoptosis, possibly through the upregulation of early apoptotic proteins such as FOS and JUN [11,12].

The *S. dulcificum* berry is currently being commercially exploited in Spain through the product DMB^®^, which consists of freeze-dried pulp and skin, produced by Medicinal Gardens S.L. (Health Registration Nº40.058621/M). This product is marketed under the brand name Baïa^®^ (www.baiafood.com, accessed on 5 November 2024), a company focused on developing and commercializing nutraceutical ingredients, functional foods, and dietary supplements with proven health benefits. DMB^®^ was authorized for commercial exploitation in 2021, following approval by the European Food Safety Authority (EFSA) under Regulation (EU) 2015/2283.

The present study evaluates the antioxidant and antitumoral potential of DMB^®^ extract in different colorectal cancer cell lines. Water was used as the extraction solvent due to its safety in cell assays, optimizing the recovery of phenolic compounds. The identified compounds were characterized using HPLC and GC-MS, providing insights for their potential therapeutic use.

## 2. Results

### 2.1. Quantification of Total Phenols and Antioxidant Capacity of the Aqueous Extract of DMB^®^

Total phenols and total flavonoids present in the aqueous extracts of DMB^®^, *P. domestica*, and *P. persica* were quantified, as shown in Table 1. The DMB^®^ extract showed the highest total phenol content (9.7 ± 0.5 mg GAE/g of dry extract), followed by *P. domestica* (4.1 ± 0.1 mg GAE/g of dry extract). In contrast, *P. persica* exhibited the lowest total phenol content (1.35 ± 0.05 mg GAE/g of dry extract), which was associated with reduced antioxidant capacity.

The antioxidant activity of the extracts was evaluated by reducing the DPPH radical. DMB^®^ showed a high DPPH reducing activity with values of 64 ± 2 µmol Trolox/g of dry extract, significantly higher than that observed in *P. domestica* (35 ± 2 µmol Trolox/g of dry extract). *P. persica*, due to its low total phenol content and considerably lower DPPH reducing capacity (4.0 ± 0.06 µmol Trolox/g of dry extract and an inhibition percentage of 2.9 ± 0.7%), was used as a negative control in the assays.

### 2.2. Chemical Characterization of DMB^®^ Aqueous Extract

The phenolic compounds from the aqueous extract of *Synsepalum dulcificum* (DMB^®^) were analyzed using HPLC-DAD and identified by comparing retention times and UV/visible spectra with the corresponding peaks from available standards. A total of twelve phenolic compounds were identified. Protocatechuic acid was the most abundant, with a concentration of 772 ± 3 µg/g dry weight (DW), followed by 174 ± 1 µg/g DW of gallic acid and 145 ± 1 µg/g DW of catechin (Table 2). The identified compounds are expressed in µg/mL and ordered by retention time, from shortest to longest. Additionally, the slope formula and the R^2^ value for each standard compound used in data interpolation are provided.

**Table 2:** Phenolic compounds from aqueous extract of DMB^®^ by HPLC.

Phenolic Compounds	Retention Time (min)	Standard Curve	Correlation Coefficient (r^2^)	Amount (µg/g WD)
Gallic acid	5.4	y = 81,204x − 5990.7	R^2^ = 0.9997	174 ± 1
Protocatechuic acid	8.6	y = 79,926x + 4177.5	R^2^ = 0.9997	771 ± 2
Catechin	10.2	y = 17,879x − 720.19	R^2^ = 0.9997	145 ± 2
Caffeic acid	12.3	y = 226,602x + 71,781	R^2^ = 0.9986	0
Syringic acid	12.6	y = 201,622x – 26,650	R^2^ = 0.9997	2.17 ± 0.01
Routine	16.21	y = 486,22x + 2371.2	R^2^ = 0.9997	0
Vanillin	17.2	y = 151,301x − 581.47	R^2^ = 0.9998	0.36 ± 0.02
Coumaric acid	17.9	y = 184,250x + 10,032	R^2^ = 0.9997	9.49 ± 0.07
Ferulic acid	18.8	y = 155,525x + 1518.5	R^2^ = 0.9998	4.3 ± 0.2
Salicylic acid	22.9	y = 24,793x − 1560.2	R^2^ = 0.9997	2.7 ± 0.2
Quercetin	25.7	y = 91,777x – 15,882	R^2^ = 0.9996	2.92 ± 0.01
Cinnamic acid	27.3	y = 234,241x + 2259.8	R^2^ = 0.9998	0.32 ± 0.05

Additionally, aqueous extracts of DMB^®^ were analyzed using gas chromatography-mass spectrometry (GC-MS) to identify potential compounds not previously reported. The identified compounds were validated by comparison with the NIST mass spectra database. The identified compounds are detailed in Table 3, ordered by retention time (RT) from lowest to highest, including their molecular formula and alphanumeric identifier (InChIKey). In the chromatographic analysis, three possible compounds were obtained for the same retention time, suggesting the presence of isomers or compounds with similar chemical structures. The identified compounds were grouped into several chemical categories: alcohols (glycerol and 2-furanmethanol), esters (2-oxopropanoic acid and hexadecanoic acid), furanic compounds (2,4-dihydroxy-2,5-dimethyl-3(2H)-furan-3-one and 5-hydroxymethylfurfural), lactones (2-oxepanone, 7-hexyl-), carbohydrates (melezitose and lactose), and fatty acids (octadecanoic acid and hexadecanoic acid, 2-hydroxy-1-(hydroxymethyl) ethyl ester).

### 2.3. Antitumor Activity

#### 2.3.1. Cell Viability

**Figure 1:**
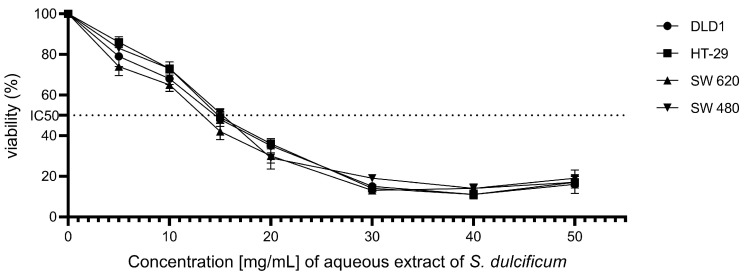
Effect of aqueous extract of DMB^®^ on the viability of colorectal cancer cells. Cell viability at different concentrations. 0, 5, 10, 15, 20, 30, 40, and 50 mg/mL of aqueous extract of DMB^®^ (*S. dulcificum* pulp + skin) in different established colorectal cell lines DLD-1, HT-29, SW480, and SW620. Dose response effects were observed in the viability of the colorectal cancer cell lines.

The effect of the aqueous extract of DMB^®^ on the cell viability of colorectal cancer cell lines DLD-1, HT-29, SW480, and SW620 was investigated using the MTT assay, a technique that measures cellular metabolic activity as an indicator of cell viability. The results revealed a significant reduction in the viability of all evaluated cell lines following exposure to the DMB^®^ extract (Figure 1). The half-maximal inhibitory concentration (IC_50_) values were determined for each cell line, yielding values of 12.3 ± 0.4 mg/mL for DLD-1, 12.5 ± 0.4 mg/mL for HT-29, 12.9 ± 0.4 mg/mL for SW480, and 11 ± 0.4 mg/mL for SW620. These results demonstrate a concentration-dependent cytotoxic activity of the DMB^®^ aqueous extract on colorectal cancer cells.

#### 2.3.2. Effect of Chemotherapy in Combination with DMB^®^

**Figure 2:**
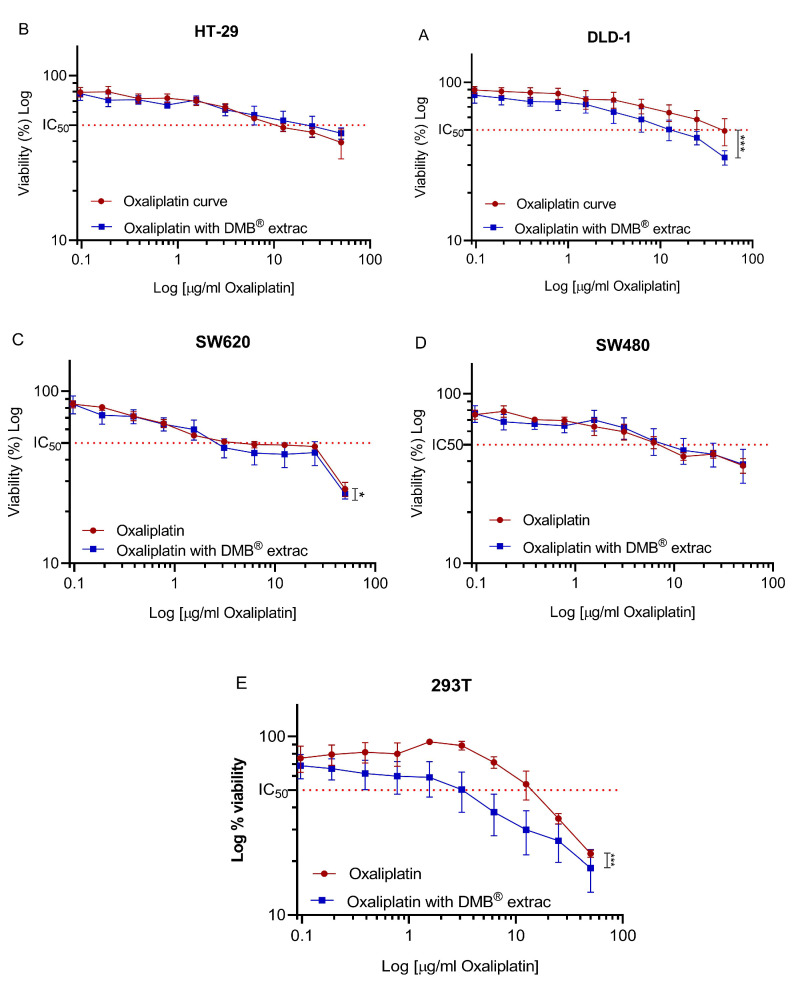
Oxaliplatin IC_50_ in different colorectal cancer DLD1, HT-29, SW480, and SW620 cell lines. Cell viability of colorectal cancer cell lines (**A**) DLD-1, (**B**) HT-29, (**C**) SW620, (**D**) SW480, (**E**) 293T against 11 different concentrations (0–50 µg/mL) of oxaliplatin and fixed concentration of aqueous extract from DMB^®^ at 7 mg/mL. The 50% inhibition concentration (IC_50_) values are calculated using the MTT assay after 72 h of exposure to the extract. Viability is reduced in a dose-dependent manner. Data are expressed as mean ± SE (n = 3), and statistical significance was determined using a two-way ANOVA test (* *p* < 0.05, *** *p* < 0.001).

To evaluate the effect of the aqueous extract of DMB^®^ in combination with oxaliplatin on cell viability, a fixed concentration of 7 mg/mL was used, equivalent to approximately half of the IC_50_ (half-maximal inhibitory concentration) value calculated for the DMB^®^ extract. For oxaliplatin, concentrations ranging from 0 to 50 µg/mL were tested through serial dilutions. The results showed a significant reduction (*p* < 0.05) in cell viability when combining oxaliplatin with DMB^®^ at the highest evaluated oxaliplatin concentration (50 µg/mL). In the DLD-1 and SW620 cell lines, cell viability decreased by up to 20% (Figure 2A) and 10% (Figure 2C), respectively, when treated with DMB^®^. In the 293T cell line, derived from non-tumor human embryonic kidney cells, a decrease in cell viability was also observed, with an IC_50_ of 15 mg/mL for oxaliplatin and 2 mg/mL for the combination of oxaliplatin and DMB^®^, respectively (Figure 2E). The IC_50_ values, along with their corresponding log IC_50_ values, are presented in Table 4. These findings suggest that the evaluated concentration of the DMB^®^ extract can enhance the cytotoxic effect of chemotherapy, improving its effectiveness in treatment.

#### 2.3.3. Cell Cycle

Since the cell viability assays showed a significant cytotoxic effect on the DLD-1 and SW620 cell lines after exposure to the DMB^®^ extract (7 mg/mL), its effect on the cell cycle was analyzed. Propidium iodide staining was performed, and samples were analyzed by flow cytometry after 72 h of treatment with DMB^®^.

**Figure 3:**
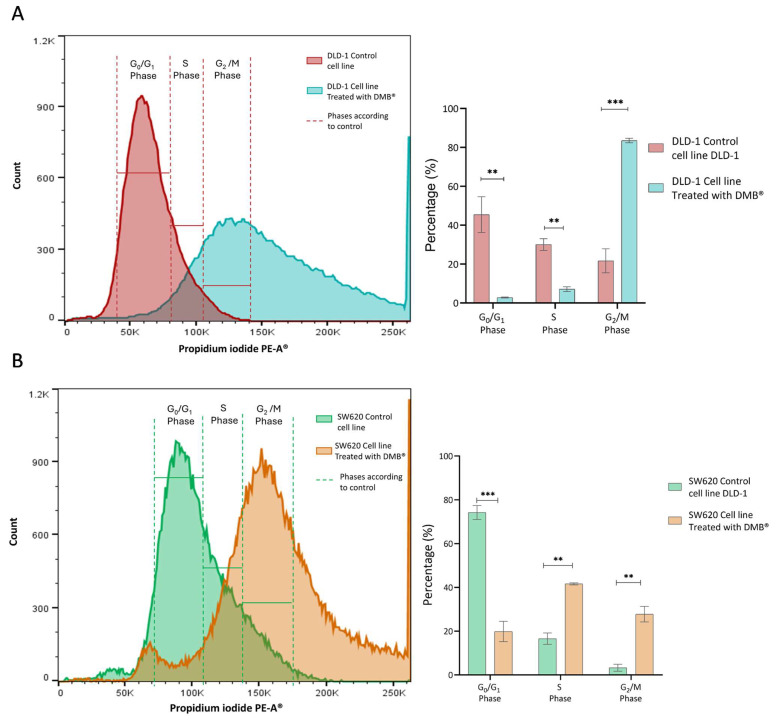
Cell cycle analysis in DLD-1 and SW620 cells treated with DMB® (7 mg/mL) for 72 h. (**A**) Representative histogram of flow cytometry analysis in DLD-1 cells, showing the distribution of cells in different phases of the cell cycle after propidium iodide staining, and in the bar graph, quantification of the percentage of DLD-1 cells in each cell cycle phase. (**B**) Representative histogram of flow cytometry analysis in SW620 cells, showing the distribution of cells in different phases of the cell cycle after propidium iodide staining, and in the bar graph, quantification of the percentage of SW620 cells in each cell cycle phase. Data are expressed as mean ± SE (n = 3) and were analyzed using a two-way ANOVA test (** *p* < 0.01, *** *p* < 0.001).

In the DLD-1 cell line (Figure 3A), the number of cells in the G_0_/G_1_ phase (*p* < 0.01) and the S phase (*p* < 0.01) decreased, while the number of cells in the G2 phase (*p* < 0.001) increased. This indicates cell arrest in the G_2_/M phase, suggesting that the extract may interfere with mitotic transition and promote cellular damage response mechanisms such as apoptosis.

In the SW620 cell line (Figure 3A), treatment with the extract also altered the cell cycle. The proportion of cells in G_0_/G_1_ (*p* < 0.001) was significantly reduced, along with an increase in those in the S phase (*p* < 0.01) and an accumulation in G_2_/M (*p* < 0.01). Unlike DLD-1, this increase in the number of S phase cells suggests possible disruption in DNA replication before the transition to G_2_/M when this cell type is exposed to treatment.

#### 2.3.4. QPCR Assay

Since the cell cycle assays using propidium iodide staining showed cell arrest in the G2/M phase, a qRT-PCR assay was performed to evaluate the effect of the DMB^®^ extract on the regulation of genes involved in apoptosis. For this purpose, the relative expression of *CASP3*, *CASP9*, *BCL2*, and *BCL2L11* was quantified in colorectal cancer DLD-1 and SW620 cells treated with 7 mg/mL of the extract for 72 h. Untreated cells were used as a control, and gene expression analysis was conducted using the 2^−ΔΔCq^ method.

**Figure 4:**
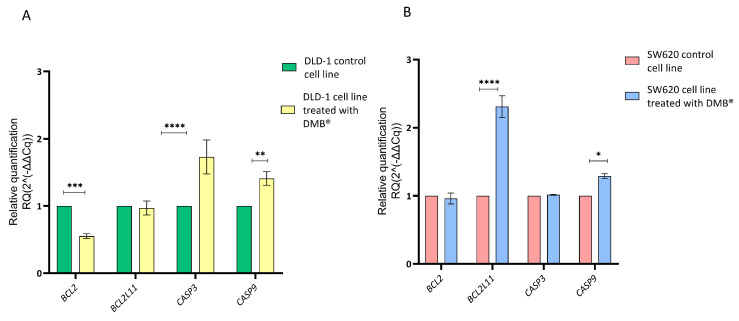
Relative gene expression in DLD-1 and SW620 cell lines after treatment with DMB^®^ (7 mg/mL) for 72 h, determined by qRT-PCR. Bar graphs show the relative expression of *BCL2*, *BCL2L11*, *CASP3*, and *CASP9*. Green and red represent control cells; yellow and blue represent DMB^®^-treated cells. (**A**) in DLD-1 cell line and (**B**) in SW620 cell line. Expression values were normalized to ACTB (beta-actin) and analyzed using the 2^−ΔΔCq^ method. Data are expressed as mean ± SE (n = 3), and statistical significance was determined using a two-way ANOVA test (* *p* < 0.05, ** *p* < 0.01, *** *p* < 0.001, **** *p* < 0.001).

The results showed that in DLD-1 cells (Figure 4A), treatment with the extract caused a significant reduction in BCL2 expression and an increase in *CASP3* and *CASP9* expression, suggesting the activation of the apoptotic pathway. In contrast, in the SW620 cell line (Figure 4B), the only significant change was an increase in *BCL2L11* expression, while *BCL2*, *CASP3*, and *CASP9* did not show significant differences compared to the control.

#### 2.3.5. Chronic Activity of DMB^®^

**Figure 5:**
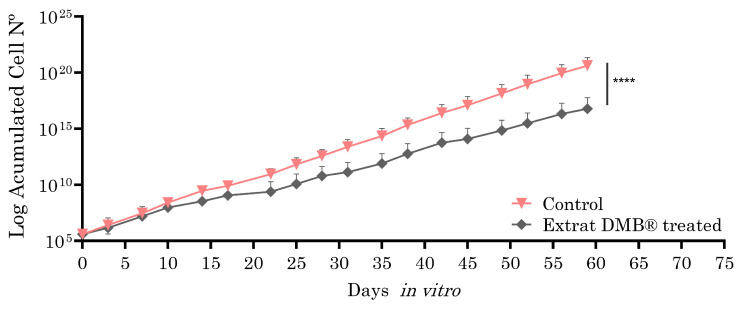
Growth curves of the SW620 cell line in and out of the presence of aqueous DMB^®^ extract. Treated cells have a significantly lower growth rate than control cells, as inferred from the growth slope of both curves. Standard error bars are shown to represent the confidence interval (two replicates). Significant differences are indicated with asterisks, determined using the Wilcoxon test (**** *p* < 0.0001).

Metastatic SW620 cells, selected for their ability to model advanced stages of colorectal cancer, such as metastasis, were exposed to the aqueous extract of DMB^®^ (0.8 mg/mL, equivalent to IC3) for two months in culture (59 days), with the aim of evaluating the long-term effects on cellular sensitivity or resistance. The choice of the SW620 cell line is due to its metastatic nature, which allows for the analysis of the extract’s impact in a context that is more representative of tumor progression. Analysis using the Wilcoxon test showed statistically significant differences, suggesting the potential of DMB^®^ to modify cellular responses over a prolonged treatment period. As shown in Figure 5, the cells treated with the DMB^®^ extract exhibited a lower proliferation rate, suggesting an inhibitory effect on metastatic SW620 cells and, consequently, increased cellular sensitivity.

**Figure 6:**
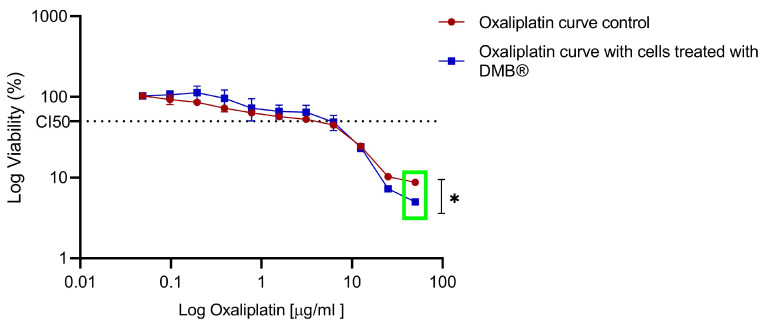
Dose–response to oxaliplatin in metastatic SW620 cells treated chronically with aqueous DMB^®^ extract. Standard error bars represent the confidence interval (n = 3). Significant differences are indicated with asterisks, denoting levels of statistical significance (* *p* ≤ 0.05), determined using the Wilcoxon test.

To compare the sensitivity observed in the DMB^®^-treated cells, both treated and untreated cells were exposed to increasing concentrations of oxaliplatin (0 to 50 µg/mL), and cell viability was assessed using the MTT assay after 72 h of exposure. In the DMB^®^-treated cells, a reduction of up to 10% in cell viability was observed at high oxaliplatin concentrations compared to the control (green box) (Figure 6).

## 3. Discussion

### 3.1. Antioxidant

The fruit of *Synsepalum dulcificum* has been widely recognized for its antioxidant properties, largely attributed to its high content in phenolic compounds [13]. In this study, the antioxidant capacity of the polyphenolic aqueous extract from DMB^®^, a commercial product derived from the lyophilized skin and pulp of *S. dulcificum*, was evaluated. Using the DPPH assay, 37% inhibition of free radicals was observed, a value similar to other berries such as *Rubus idaeus* (23.3%), *Fragaria × ananassa* (15%), and *Ribes nigrum* (42%) [14]. However, this activity differed from that reported by Haddad et al., 2020 [15], who studied aqueous and ethanolic extracts of *S. dulcificum*, obtaining values of 67% and 79%, respectively, indicating that the pulp has greater antioxidant capacity. However, DMB^®^ demonstrated significantly higher antioxidant capacity compared to other fruits, such as *Prunus domestica* (red plum), with nearly double the free radical scavenging ability, highlighting its potential for clinical applications and its commercial interest.

### 3.2. Extraction Phenolic

The major phenolic compounds in the aqueous extract of DMB^®^ were identified using HPLC. Protocatechuic acid was the most abundant compound (771 ± 2 µg/g DW), followed by gallic acid (174 ± 1 µg/g DW) and catechin (145 ± 2 µg/g DW), all of which are well known for their antioxidant and antineoplasic effects. These results are in agreement with previous studies that also detected gallic acid and catechin in aqueous fruit extracts, including those of *S. dulcificum* (L Du et al., 2014; Haddad et al., 2020 [15,16]). However, this is the first time that considerable amounts of protocatechuic acid (3,4-dihydroxybenzoic acid) are found in this plant. This acid is a metabolite with significant impact on human health, with anti-inflammatory, neuroprotective, anticardiotoxic, and anticancerogenic activities [17,18], which could explain the cytotoxic effects of these extracts on colorectal cancer cells, as found in this study. It is important to remark that one stereoisomer of this compound has been previously identified with UHPLC-Q-TOFMS in aqueous extracts of pulp/skin [19].

### 3.3. Analysis HPLC/GC-MS

The analysis of compounds extracted from DMB^®^ using gas chromatography-mass spectrometry (GC-MS) has shown a wide range of chemical compounds, highlighting the complexity of the aqueous extract of DMB^®^. Among the most relevant compounds, p-Dioxane-2,3-diol (RT 5.14) and melezitose (RT 15.67 min) are notable for their roles as cryoprotectants, flavor enhancers, and potential surfactants [20]. Additionally, l-(+)-ascorbic acid 2,6-dihexadecanoate (RT 20.11 min) was identified as a potential anti-inflammatory agent [21], and 1-glycerol palmitate (RT 24.26 min) was recognized for its anticancer properties [19,22]. Glycerin and hexadecanoic acid were also identified, both widely used in the cosmetic and pharmaceutical industries for their anti-inflammatory, antioxidant, moisturizing, and emollient properties [23,24]. Furthermore, 2,5-dimethylfuran-3,4(2H,5H)-dione and erythritol exhibit antioxidant properties, making them useful for the food industry in terms of production [25,26,27]. Finally, 3-amino-2-oxazolidinone and propanoic acid demonstrate pharmaceutical potential due to their antimicrobial characteristics [25,26,27].

### 3.4. Cellular Viability

DMB^®^ induced a dose-dependent reduction in cell viability, achieving a decrease in cell proliferation of over 50%, which allowed the determination of the corresponding IC_50_ values. Previous studies evaluated the DMB^®^ extract in the DLD-1 cell line but were unable to determine the IC_50_ value due to insufficient concentrations. However, for the HT-29 cell line, an IC_50_ of 0.05 mg/mL was determined, which is 26% lower than the 13 mg/mL found in our study, possibly due to differential cellular sensitization based on each line’s susceptibility to the extract and its characteristics. The method of preparation and preservation of the extract also influences the bioactive molecules present and should be standardized to optimize its future biomedical applications [12,28]. Given the effect of DMB^®^ on cell lines, it can be inferred that the extract interferes with essential cellular processes involved in tumor cell proliferation, suggesting its potential as a candidate for alternative therapies or, perhaps more interestingly, its potential as a complementary therapy in the treatment of specific cancers by improving the efficiency of current treatments, lowering doses in patients and thus minimizing unwanted side effects in these patients.

In addition to the effects observed in tumor cell lines, the DMB^®^ extract was evaluated in healthy 239T cells, where it exhibited a dose–response behavior like that of chemotherapeutic agents. This finding suggests that, like conventional treatments, certain natural extracts can exert cytotoxic effects on both cancerous and healthy cells. Previous studies have documented the cytotoxic effects of natural bioactive compounds on non-cancerous cells, highlighting the importance of adjusting concentrations to minimize side effects in non-tumoral tissues. An example of this phenomenon is seen with extracts of *Sambucus nigra* L. berries, which show cytotoxicity in the non-transformed NCM460 colon cell line [29].

The antiproliferative effects observed in colorectal cancer cells may be related to the antioxidant capacity of the DMB^®^ extract, which contains phenolic compounds that reduce reactive oxygen species (ROS), a relevant factor in the context of cancer, as a pro-oxidant environment promotes tumor proliferation and progression. Compounds such as protocatechuic acid [18] and gallic acid [30] present in DMB^®^ act as antioxidants by neutralizing ROS and simultaneously modulating signaling pathways involved in apoptosis and growth inhibition, thereby contributing to cytotoxicity in tumor cells and interfering with the tumor microenvironment. It is important to note that DMB^®^ is a natural extract marketed as a dietary supplement, not a purified active ingredient, meaning it contains multiple bioactive molecules that may exert effects on various signaling pathways. These effects—including those studied, such as antiproliferative and antioxidant activities, and others not investigated here—may present synergies and antagonisms that require further study to fully understand. However, given that the extract is commercialized and consumed as a dietary supplement, identifying physiological responses and potential health benefits from consuming the full DMB^®^ extract is relevant.

### 3.5. Chemotherapy

Oxaliplatin, the chemotherapy drug used in the experiments, induces oxidative stress at the neuronal level, leading to neurotoxicity in patients. Mitigating this oxidative stress and its concomitant effects in patients undergoing chemotherapy treatment is a future application of the extract for both pathways: neurotoxicity protection and synergistic antiproliferative effects with chemotherapy, which we aim to explore further. Regarding the non-complementary nature of the observed effects, both antiproliferative and antioxidant, there are already dietary supplements, including proteins, that exhibit these two combined effects. Curcumin alters distinct molecular pathways in breast cancer subtypes revealed by integrated miRNA/mRNA expression analysis [31].

Furthermore, many pro-inflammatory mediators, such as prostaglandins, prostacyclins, thromboxanes, leukotrienes, and platelet-activating factors, are involved in proliferation, migration, invasion, and metastasis. The described extract shows effects on both physiological processes, proliferation, and inflammation, with a regulatory effect on both. For example, corosolic acid, an anti-inflammatory and anticancer molecule, has been proposed for future use in developing a new drug [32].

### 3.6. Cell Cycle and qRT-PCR

The reduction in *BCL2* expression and the increase in *CASP3* and *CASP9* observed in the DLD-1 and SW620 cell lines after exposure to the extract demonstrate that DMB^®^ induces apoptosis. Presence of BCL2 protein, dependent on caspase-3, promotes the release of cytochrome c, which forms the apoptosome protein complex and triggers the activation of caspase-9. The activation of caspase-9 subsequently activates caspase-3, initiating the pro-apoptotic caspase cascade [33]. This modulation affects the cell cycle, leading to cell cycle arrest in the S and G_2_/M phases.

Previous studies have shown that extracts from *Synsepalum dulcificum* can regulate the expression of *C-FOS* and *C-JUN* in colorectal cancer cells [28]. The resulting proteins, which are overrepresented in cancer cells, are part of the AP-1 complex, which is involved in the regulation of the cell cycle and apoptosis through arrest in the G_2_/M phase [34]. This activation of C-FOS and *C-JUN* expression could be the pathway through which DMB^®^ exerts its effect, promoting cell cycle inhibition in the G_2_/M phase, as observed in the DLD-1 cell line. On the other hand, Zhang et al. (2023) demonstrated that phenolic extracts from *Synsepalum dulcificum* inhibit the mTOR (mammalian target of rapamycin) pathway in colorectal cancer cells. Since mTOR is a key regulator of cell growth and cycle progression, its inhibition leads to arrest in the S phase, which could explain the cell cycle interruption observed in the SW620 cell line in response to the treatment [12].

The effect of the extract on the cell cycle of DLD-1 and SW620 cell lines could enhance the action of chemotherapeutic agents. In DLD-1, the extract induces arrest in the G_2_/M phase [35], making it a potential candidate for combination with oxaliplatin, an agent that causes DNA damage and halts the cell cycle at this phase. Furthermore, our experiments have shown a significant reduction in DLD-1 cell viability when DMB^®^ treatment is combined with oxaliplatin, suggesting a possible synergistic effect between both compounds. In SW620, the arrest in the S phase suggests that its combination with 5-fluorouracil (5-FU) [36], an antimetabolite that inhibits DNA replication at this phase, could increase cytotoxicity in this cell line.

### 3.7. Chronic Assays

In chronic assays, prolonged treatment with the aqueous extract of DMB^®^ not only maintained its cytotoxic effect but also progressively reduced cell proliferation. These results are particularly relevant, as one of the main challenges in treatment with drugs like oxaliplatin is the development of long-term tumor resistance, which compromises its therapeutic efficacy. The behavior observed with the DMB^®^ extract is consistent with previous studies on antioxidant treatments based on natural compounds extracted from fruits like berries [37] and suggests that this extract could offer a complementary strategy to mitigate such resistance.

The fact that the extract not only maintains its cytotoxic activity but also reduces tumor proliferation over time highlights its potential as an adjuvant in prolonged therapies. If, as we postulate, the adjuvant use of the extract could contribute to reducing the drug dose necessary to achieve an effective therapeutic outcome, thus minimizing the side effects associated with the drug, this would imply a major step forward in cancer treatment. It is crucial to further verify this effect and to continue investigating the *S. dulcificum* extract as a complementary agent to standard chemotherapy regimens. We consider the study of this effect in vivo, by use of experimental models, to be of particular interest, paying particular attention to the effect on tumor-initiating cells, which would put the focus on preventing tumor recurrence and reducing resistance to current treatments.

In this work, after chronic treatments, a second cell viability assay was conducted in combination with oxaliplatin. A reduction in cell viability was observed at high oxaliplatin concentrations (40–50 µg/mL), with a decrease in cell proliferation of up to 10%. Chronically exposed cells show increased resistance to oxaliplatin, suggesting a positive effect of the aqueous extract of *Synsepalum dulcificum* in combination with chemotherapy, which is very interesting in addition to the previously discussed acute effect. In other words, both the concentrations of use of the extract and the application times should be precisely adjusted to maximize the desired therapeutic effects and minimize the occurrence of unwanted adverse effects. This effect could increase the efficacy of oxaliplatin in resistant cells. In future research, it would be important to examine the proteins directly related to resistance to determine the cellular responses that are generated, as reported in other studies [38].

## 4. Materials and Methods

### 4.1. Origin of Plant Materials

The commercial product DMB^®^, composed of the processed pulp and skin of *Synsepalum dulcificum*, was used. The fruit was harvested and lyophilized in 2018 in Ghana (Africa). The resulting powder was stored in hermetically sealed aluminum sachets and kept at 4 °C. The material was provided by the company Medicinal Gardens S. L. (Baïa Food Co., Madrid, Spain).

Three commercially obtained fruits originally harvested in Spain were also selected for comparison purposes. *Prunus domestica* (red plum) and *Vaccinium myrtillus* (blueberry) were used as positive controls for antioxidant assays and *Prunus persica* var. platycarpa (donut peach) as a negative control due to their respective high and low antioxidant capacity. These fruits were washed with distilled water and later homogenized with an immersion blender (Qilive Q.5336, Croix, France), including only the pulp and skin. The blend was subsequently lyophilized (BIOBASE BK-FD10 series, Jinan, Shandong, China). After 48 h, the resulting samples were pulverized with an immersion blender and stored in a drying chamber at room temperature.

### 4.2. Total Phenol Extraction

The extraction was performed at a 1:10 *w*/*v* ratio using Milli-Q water, under continuous magnetic stirring for 1 h at room temperature. These were then centrifuged for 20 min at 8500× *g*, room temperature. The resulting supernatant was then collected and stored at −20 °C and protected from light. Three independent extractions were performed. The specifications of all the compounds used for phenol extraction are summarized in Table S1 (including CAS numbers, suppliers, and purities).

### 4.3. Determination of Total Phenolic Content (TPC)

Total phenolic content (TPC) was determined using the modified Folin–Ciocalteu method [39]. A 20% (*w*/*v*) Na2CO3 solution (Labkem, Barcelona, Spain) was previously prepared by heating until boiling and letting it rest for 24 h. The extracts were prepared at a concentration of 1 mg/mL with distilled water. Then, 50 μL of Folin–Ciocalteu (PanReac AppliChem, Barcelona, Spain) reagent was added to the previously prepared extract. After 15 min of reaction, 150 μL of Na_2_CO_3_ solution was added, and the mix was incubated for 2 h in darkness. The absorbance was measured at 765 nm in a visible UV spectrophotometer (BIOBASE-EL 10A, Jinan, Shandong, China), using each corresponding solvent as blanks. The results were expressed as mg equivalent of gallic acid (GAE) per gram of sample in dry weight (DW) using Equation (1):

(1) $$mg\;GAE/g\;DW=\left(eq\frac{mg}{ml}\times \frac{mL}{g}\right)\times DF/1000$$

The specifications of all the compounds used for determination of total phenolic content are summarized in Table S2 (including CAS numbers, suppliers, and purities).

### 4.4. Determination of Antioxidant Activity Assays (TEAC) by DPPH•

Antioxidant activity assays were performed in 96-well plates [40]. Samples were diluted to 5 mg/mL in distilled water. Then, 150 μL of 0.5 mM DPPH (2,2-difenil-1-picrylhydrazyl, Sigma, St. Louis, MO, USA) was added. After 30 min of incubation in darkness, absorbance was measured at 517 nm in a UV visible spectrophotometer (BIOBASE-EL 10A, Jinan, Shandong, China), using each corresponding solvent as a blank. The results were expressed as µmol equivalent of Trolox (TEAC) per gram of sample in dry weight (DW) using Equations (2) and (3) as follows:

% inhibition = ((Ac (control) − As (sample))/(Ac (control))) × 100(2)

where Ac (control) is DPPH absorbance without extract; and As (sample) is DPPH absorbance with extract.

µmol Trolox g DW = (% inhibition eq uM trolox × L)/g(3)

where % inhibition is the interpolated DPPH inhibition percentage from the Trolox standard curve; L is the extraction volume; and g is the amount of dry sample used for extraction.

The specifications of all the compounds used for antioxidant activity assays are summarized in Table S3 (including CAS numbers, suppliers, and purities).

### 4.5. Determination of Phenolic Composition by HPLC Analysis

The quantification of the phenolic compounds found in DMB^®^ was performed by high-performance liquid chromatographic (HPLC) analysis on a JASCO 4000 Series HPLC system (JASCO, Tokyo, Japan) equipped with a photo diode array (DAD) detector, using a Fortis C18 column (250 mm × 4.6 mm, 5 µm) at room temperature.

Detection and identification of the phenolic compounds were performed using a DAD detector at the following wavelengths: 271 nm for the detection of gallic acid, protocatechuic acid, and syringic acid; 282 nm for the detection of catechin, vanillin, salicylic acid, and cinnamic acid; 323 nm for the detection of caffeic acid, p-coumaric acid, and trans-ferulic acid; and 370 nm for the detection of rutin and quercetin.

The compounds were separated with a gradient elution using different ratios of 2% aqueous acetic acid solution (*v*/*v*) (A) and acetonitrile (B). The gradient method conditions were as follows: initially 10% of phase B; then linear increase from 10% to 21% of B during the first 6 min; maintain the conditions until 12th min; linear increase in B to 35% from 12 to 15 min; maintain the conditions until 22nd min; linear increase in B to 50% from 22 to 28 min; and finally, linear decrease to 10% of B between 28 and 32 min using a flow rate of 1 mL/min. Re-equilibration of the column was carried out using the starting conditions for 6 min before the next assay. Total analysis per sample was performed in 38 min.

The quantification of the extraction of the target compounds was measured by the integration of the peak area and calculated using the calibration curves prepared with standard solutions for each compound. The results, expressed as µg of phenolic compound per g DW, were obtained using Equation (4):

PC ((µg)⁄(g DW)) = ([Phenolic compounds])/(m sample DW) · V solvent(4)

where [phenolic compounds] is the concentration of the phenolic compounds expressed in µg/L, measured by HPLC and calculated with the calibration curve; V solvent is the amount of solvent used during the extraction expressed in L; and m sample DW is the amount of dry sample used expressed in g.

The specifications of all the compounds used for HPLC analysis are summarized in Supplementary Materials Table S4 (including CAS numbers, suppliers, and purities).

### 4.6. GC/MS Chromatographic

Thermo TSQ DUO equipment (Thermo Fischer Scientific, Waltham, MA, USA) was used for the separation and identification of compounds. Helium served as the mobile phase with a flow rate of 1 mL/min, and the injector temperature was maintained at 220 °C. The RTX5-ms column was utilized under the following conditions: an initial temperature of 40 °C for 4 min followed by a temperature increase to 270 °C at a rate of 12 °C/min, which was held for 7 min. The mass spectrometer system was operated in electron ionization (EI) mode at 70 eV. The ion source and transfer line temperatures were both 280 °C, and interpretation of the detected masses was through NIST.

### 4.7. Cell Assays

Colorectal cancer cell lines DLD-1 (CCL-221), HT-29 (HTB-38), SW480 (CCL-228), and SW620 (CCL-227) from the American Type Culture Collection (ATCC, Manassas, VA, USA) were used. The cells were cultured in Dulbecco’s modified Eagle’s medium (DMEM-High Glucose, Dominique Dutscher, Brumath, France) with addition of 10% fetal bovine serum (FBS, PAN Biotech, Aidenbach, Germany), 1% penicillin/streptomycin (Corning, New York, NY, USA), and 2 mM L-glutamine (FBS, PAN Biotech, Aidenbach, Germany) at 37 °C with 5% CO2 in an incubator (Series II water Jacker, Thermo Scientific, Waltham, MA, USA).

The dose–response assays were established in 96-well plates. The cells were seeded in a volume of 100 µL at a concentration of 100,000 cells/mL and incubated for 24 h. The aqueous extracts of DMB^®^ were then added in the range of concentrations 0, 5, 10, 15, 20, 30, 40, and 50 mg/mL, and these were incubated at 37 °C with 5% CO_2_.

The aqueous extracts of DMB^®^ were added at 7 mg/mL and increasing concentrations of oxaliplatin [0 till 50 µg/mL] and these incubated at 37 °C with 5% CO_2_. DMEM culture medium was used as a negative control.

After 72 h, cell viability was detected with the thiazolyl blue tetrazolium bromide assay (MTT, BioChem, PanreacApplichem, Barcelona, Spain). The medium was removed, and 0.5 mg/mL MTT solution was added. Then, the plate was incubated for 4 h at 37 °C, and finally, the solution was removed and the formed formazan crystals resuspended in dimethyl sulfoxide (DMSO, Labkem, Barcelona, Spain). The detection of absorbance was measured at 546 nm (BIOBASE-EL 10A, Shandong, China). These assays consisted of 3 independent biological replicates with 4 technical replicates each.

### 4.8. Cell Cycle: Propidium Iodide

DLD-1 and SW620 cells were treated with 7 mg/mL of DMB^®^ extract for 72 h, while untreated cells were used as a control. Cells were detached using trypsin for 5 min at 37 °C and collected by centrifugation at 300× *g* for 5 min. The cells were resuspended in 500 µL of PBS 1X and incubated with 5 µL of propidium iodide solution at a concentration of 50 µg/mL. The mixture was incubated at room temperature in the dark for 15 min to allow dye incorporation into the DNA. Subsequently, the cells were analyzed by flow cytometry using a 488 nm excitation laser. Fluorescence distribution histograms were obtained, and the percentages of cells in each cell cycle phase (G_0_/G_1_, S, and G_2_/M) were analyzed using FlowJo™ v10.8 software (BD Life Science, Franklin Lakes, NJ, USA).

### 4.9. qRT-PCR

#### 4.9.1. RNA Extraction and cDNA Synthesis

DLD-1 and SW620 cells were treated with 7 mg/mL of DMB^®^ extract for 72 h, while untreated cells were used as a control. Cells were detached using trypsin for 5 min at 37 °C and collected by centrifugation at 300× *g* for 5 min. Total RNA from cultured cells was extracted using the RNAeasy mini kit (Qiagen, Hilden, Germany), following the manufacturer’s instructions. RNA concentration and purity were measured using spectrophotometry (BioNova, Fremont, CA, USA), ensuring that the 260/280 ratios were between 1.8 and 2.0, and stored at −80 °C until use.

cDNA synthesis was performed using the Revertaid first-strand cDNA synthesis kit (Thermo Scientific™). The reaction was carried out using a CFX Opus system Real-Time cycler (Bio-Rad, Hercules, CA, USA) under the following conditions: a mixture containing 1 µg of RNA, 1 µL of random hexamers (10 mm), and H_2_O Milli-Q up to 12 µL was incubated at 70 °C for 5 min. Subsequently, 4 µL of 5× buffer, 1 µL of ribonuclease inhibitor (20 U/µL), 2 µL of DNTPs (10 mmol), and 1 µL of reverse transcriptase (12 U/µL) were added to each tube. The reaction mixture was incubated at 25 °C for 5 min, 42 °C for 1 h, and inactivated at 70 °C for 10 min. All cDNA samples were stored at −20 °C until use.

#### 4.9.2. Quantitative PCR (qPCR)

The quantitative PCR (qPCR) reaction was performed using SSOAdvanced™ universal SYBR^®^ green supermix (Bio-Rad) according to the manufacturer’s instructions. The primer sequences were as follows: (1) *BCL2L11*: 5′-ATGCAAGGAGGGTATTTTTG-3′ (forward) and 5′-CGTAACAGTCGTAAGATAACC-3′ (reverse); (2) *BCL2L11*: 5′-GATTGTGGCCTTCTTTGAG-3′ (forward) and 5′-GTTCCACAAAGGCATCC-3′ (reverse); (3) *CASPASE-3*: 5′-TACCAGTGGAGGCCGACTTC-3′ (forward) and 5′-GCACAAAGCGACTGGATGAAC-3′ (reverse); (4) *CASPASE-9*: 5′-GGACATCCAGCGGGCAGG-3′ (forward) and 5′-TCTAAGCAGGAGATGAACAAAGG-3′ (reverse); (5) *ACTB* (actin beta human): 5′-GACGACATGGAGAAAATCTG-3′ (forward) and 5′-ATGATCTGGGTCATCTTCTCG-3′ (reverse). As a reference gene (housekeeping gene), β-ACTIN was used.

A volume of 0.3 µL of each primer at a concentration of 10 pmol/µL was used, along with 10 ng of cDNA per well, in a total volume of 10 µL. cDNA samples were analyzed in triplicate on a 96-well plate, sealed with adhesive seals, using the Roche Lightcycler 480 thermocycler (Roche, Basel, Switzerland). The amplification protocol consisted of an initial phase of 10 min at 95 °C, followed by 45 cycles under the following conditions: 15 s at 95 °C, 1 min at 60 °C, and 10 s at 72 °C. For the calculation of normalized relative mRNA expression, the 2^−ΔΔcq^ method was used.

### 4.10. Chronification of Cells

#### 4.10.1. Assay Chronification

The chronification of the SW620 metastatic colorectal cancer cell line was carried out by adding DMB^®^ at 0.8 mg/mL (equivalent to IC3) for 59 days, during which the cumulative growth rate was measured using a LUNA-II™ automatic cell counter (Logos Biosystem, Anyang, South Korea). Subsequently, the cells were exposed to oxaliplatin (0, 0.04, 0.097, 0.19, 0.39, 0.78, 1.56, 3.125, 6.25, 12.5, 25, 50 µg/mL) and compared with non-chronified cells.

#### 4.10.2. Tumor Biomarker Analysis: Immunostaining

The expression of different biomarkers was analyzed by flow cytometry. SW620 cells were collected using trypsin for 5 min at 37 °C and centrifuged at 300× *g* for 5 min. The cells were incubated with the corresponding antibody in a volume of 50 μL for 15 min at room temperature, in darkness, and under agitation. The required dilution and the commercial brand of each antibody are shown in Table S3. Subsequently, the antibody was inactivated with 500 µL of PBS 1X and removed by centrifugation at 400× *g* for 5 min, and cells were fixed with GLYO-FIXX™ (Thermo Scientific) at a 1:1 ratio with PBS 1X-BSA at 0.5%, obtaining a final volume of 50 μL per tube. Finally, the cells were analyzed using a FACS CANTO II cytometer (Becton Dickinson, Franklin Lakes, NJ, USA). All data were processed and analyzed using FLOWJO™ v10.8 software (BD Life Sciences).

### 4.11. Statistical Analysis

One-way analysis of variance (ANOVA) was used to determine the differences between three groups. Wilcoxon tests were used to compare the statistical differences between two paired groups and the Friedman test to compare three or more paired groups. A *p*-value < 0.05 was employed in all of the tests. Statistical analysis was performed using GraphPad Prism 8 (GraphPad Software Inc., San Diego, CA, USA), and statistical significance is indicated as * *p* ≤ 0.05; ** *p* ≤ 0.01; *** *p* ≤ 0.001; **** *p* ≤ 0.0001, which refers to the condition presenting maximum extraction output.

## 5. Conclusions

In conclusion, it is important to highlight that the levels of bioactive compounds present in the DMB^®^ extract (pulp and skin of *Synsepalum dulcificum*) are influenced by various factors, such as the plant’s growing conditions (soil, climate, and exposure to stress factors). Additionally, both the extraction method and storage conditions directly affect its potency. In our case, we used water as a solvent, but it is important to consider that the type of solvent used may also influence the extract’s biological activity, which should be carefully considered in future studies.

Analyses using HPLC and GC-MS allowed us to identify various molecules of industrial interest, which could be involved in the high antioxidant potential demonstrated by this plant. This antioxidant power could be related to the anticancer properties observed. In our experiments, combining DMB^®^ with the chemotherapeutic agent oxaliplatin resulted in a significant reduction in the drug dose needed to induce cytotoxic effects in DLD-1 and SW620 cell lines. Similarly, the chronic administration of DMB^®^ in the metastatic SW620 cell line showed sensitization to treatment, suggesting that the extract could be a potential adjuvant in anticancer therapies.

Nevertheless, more comprehensive studies in animal models are necessary to determine its efficacy, optimal administration routes, and potential long-term toxic effects before moving towards clinical trials in humans.

Cell cycle analyses showed that DMB^®^ induces arrest in the G_2_/M phase in the DLD-1 cell line and in the S phase in the SW620 cell line, and its mechanism of action could be related to cell cycle regulation and inhibition of cell proliferation. qRT-PCR assays demonstrated that treatment with DMB^®^ in DLD-1 induces an increase in the expression of *CASP3* and *CASP9* and a decrease in *BCL2*, indicating the activation of the intrinsic apoptotic pathway. In contrast, in SW620, *BCL2L11* expression was increased without significant changes in *CASP3* and *CASP9*, suggesting that apoptosis in this cell line may be regulated by alternative mechanisms.

## Figures and Tables

**Table 1:** Analysis of total phenols and antioxidant capacity of aqueous extracts of DMB^®^ and control fruits.

			DPPH	
	Total Phenols (mg GAE/mg Dry Extract)	Total Flavonoids (mg QE/mg Dry Extract)	µmol Trolox/gr Dry Extract	% Inhibition
DMB^®^	9.7 ± 0.5	3.3 ± 0.2	64 ± 2	37 ± 1
*P. domestica*	4.1 ± 0.1	2.3 ± 0.4	35 ± 2	20 ± 1
*P. persica*	1.35 ± 0.05	2.3 ± 0.3	4.0 ± 06	2.9 ± 0.7

**Table 3:** Compounds from aqueous extract of DMB^®^ by CG-MS.

Peak	Retiention Time	Option 1	Molecular Formula	InChIKey	Option 2	Molecular Formula	InChIKey	Option 3	Molecular Formula	InChIKey
1	5.14	Glycerin	C_3_H_8_O_3_	PEDCQBHIVMGVHV-UHFFFAOYSA-N	3-Methoxy-2,2-dimethyloxirane	C_5_H_10_O_2_	FPKWGRVMLLIFSY-UHFFFAOYSA-N	p-Dioxane-2,3-diol	C_4_H_8_O_4_	YLVACWCCJCZITJ-UHFFFAOYSA-N
2	6	Propanoic acid, 2-oxo-, methyl ester	C_4_H_6_O_3_	CWKLZLBVOJRSOM-UHFFFAOYSA-N	3-Amino-2-oxazolidinone	C_3_H_6_N_2_O_2_	KYCJNIUHWNJNCT-UHFFFAOYSA-N	Ethyl acetoxycarbamate	C_5_H_9_NO_4_	PWDCNDKCDGSDGY-UHFFFAOYSA-N
3	7.27	2-Furanmethanol	C_5_H_6_O_2_	XPFVYQJUAUNWIW-UHFFFAOYSA-N	3-Furanmethanol	C_5_H_6_O_2_	STJIISDMSMJQQK-UHFFFAOYSA-N	Methylenecyclopropanecarboxylic acid	C_5_H_6_O_2_	QJUQASYVMKRUMN-UHFFFAOYSA-N
4	9.38	2,4-Dihydroxy-2,5-dimethyl-3(2H)-furan-3-one	C_6_H_8_O_4_	JYMIRUWYSKOKRU-UHFFFAOYSA-N	4H-Pyran-4-one, 2,3-dihydro-3,5-dihydroxy-6-methyl-	C_6_H_8_O_4_	JYMIRUWYSKOKRU-UHFFFAOYSA-N	N-(1-Methoxycarbonyl-1-methylethyl)-4-methyl-2-aza-1,3-dioxane	C_9_H_17_NO_4_	VWGVVWKWTUQAIE-UHFFFAOYSA-N
5	10.65	2,5-Dimethylfuran-3,4(2H,5H)-dione	C_6_H_8_O_3_	PUVDDHUFFRRFMN-UHFFFAOYSA-N	Furaneol	C_6_H_8_O_3_	INAXVXBDKKUCGI-UHFFFAOYSA-N	2,5-Piperazinedione, 3-methyl-	C_5_H_8_N_2_O_2_	ICCHEGCKVBMSTF-UHFFFAOYSA-N
6	11.94	4H-Pyran-4-one, 2,3-dihydro-3,5-dihydroxy-6-methyl-	C_6_H_8_O_4_	VOLMSPGWNYJHQQ-UHFFFAOYSA-N	2,4-Dihydroxy-2,5-dimethyl-3(2H)-furan-3-one	C_6_H_8_O_4_	JYMIRUWYSKOKRU-UHFFFAOYSA-N	2-Propyl-tetrahydropyran-3-ol	C_8_H_16_O_2_	SMMBPJGNKCWQPY-UHFFFAOYSA-N
7	12.96	5-Hydroxymethylfurfural	C_6_H_6_O_3_	NOEGNKMFWQHSLB-UHFFFAOYSA-N	Furan, 2,3-dihydro-4-(1-methylpropyl)-, (S)-	C_8_H_14_O	UQEZSQDDLZCNRH-ZETCQYMHSA-N	4-Mercaptophenol	C_6_H_6_OS	BXAVKNRWVKUTLY-UHFFFAOYSA-N
8	13.36	2-Oxepanone, 7-hexyl-	C_12_H_22_O_2_	FRTMRFCNTDDSOB-UHFFFAOYSA-N	Caprolactam	C_6_H_11_NO	JBKVHLHDHHXQEQ-UHFFFAOYSA-N	2-Oxepanone, 7-butyl-	C_10_H_18_O_2_	YKVIWISPFDZYOW-UHFFFAOYSA-N
9	13.69	Octanamide, N-(2-mercaptoethyl)-	C_10_H_21_NOS	JLZORHOCSVVPHT-UHFFFAOYSA-N	Maltose	C_12_H_22_O_11_	GUBGYTABKSRVRQ-QUYVBRFLSA-N	6-oxoheptanoato de metilo	C_8_H_14_O_3_	BSBYQAYWPXHLPQ-UHFFFAOYSA-N
10	15.67	Melezitose	C_18_H_32_O_16_	QWIZNVHXZXRPDR-WSCXOGSTSA-N	Maltose	C_12_H_22_O_11_	GUBGYTABKSRVRQ-QUYVBRFLSA-N	Lactose	C_12_H_22_O_11_	GUBGYTABKSRVRQ-DCSYEGIMSA-N
11	16.42	2,6-Diamino-4-cyclohexyl-4H-thiopyran-3,5-dicarbonitrile	C_13_H_16_N_4_S	RBAOYVGXTOUWKG-UHFFFAOYSA-N	Cyclohexane, 1,4-dimethyl-2-octadecyl-	C_26_H_52_	IYAUESUIHMJWPO-UHFFFAOYSA-N	Octa-2,6-diene, 2,7-dimethyl-4-phenylthio-	C_16_H_22_S	OTLKSJYKRHNQQB-UHFFFAOYSA-N
12	19.82	Hexadecanoic acid, methyl ester	C_17_H_34_O_2_	FLIACVVOZYBSBS-UHFFFAOYSA-N	Pentadecanoic acid, 13-methyl-, methyl ester	C_17_H_34_O_2_	FRGDXZRZDAJTOU-UHFFFAOYSA-N	Pentadecanoic acid, 14-methyl-, methyl ester	C_17_H_34_O_2_	WAKCWJNDXBPEBP-UHFFFAOYSA-N
13	20.11	l-(+)-Ascorbic acid 2,6-dihexadecanoate	C_38_H_68_O_8_	TUYRNAGGIJZRNM-LBHUVFDKSA-N	n-Hexadecanoic acid	C_16_H_32_O_2_	IPCSVZSSVZVIGE-UHFFFAOYSA-N	Pentadecanoic acid	C_15_H_30_O_2_	WQEPLUUGTLDZJY-UHFFFAOYSA-N
14	21.68	Octadecanoic acid	C_18_H_36_O_2_	QIQXTHQIDYTFRH-UHFFFAOYSA-N	Octadecanoic acid, 2-(2-hydroxyethoxy)ethyl ester	C_22_H_44_O_4_	PWVUXRBUUYZMKM-UHFFFAOYSA-N	L-Ascorbic acid, 6-octadecanoate	C_24_H_42_O_7_	LITUBCVUXPBCGA-WMZHIEFXSA-N
15	24.26	Hexadecanoic acid, 2-hydroxy-1-(hydroxymethyl)ethyl ester	C_19_H_38_O_4_	BBNYCLAREVXOSG-UHFFFAOYSA-N	Glycerol 1-palmitate	C_19_H_38_O_4_	QHZLMUACJMDIAE-UHFFFAOYSA-N	Hexadecanoic acid, 1-(hydroxymethyl)-1,2-ethanediyl ester	C_35_H_68_O_5_	JEJLGIQLPYYGEE-UHFFFAOYSA-N

**Table 4:** IC_50_ in µg/mL of oxaliplatin and oxaliplatin with DMB^®^ in colorectal cancer cell lines DLD1, HT-29, SW480, and SW620.

	DLD-1		HT-29		SW620		SW480		293T	
	Oxaliplatin	Oxaliplatin + DMB^®^	Oxaliplatin	Oxaliplatin + DMB^®^	Oxaliplatin	Oxaliplatin + DMB^®^	Oxaliplatin	Oxaliplatin + DMB^®^	Oxaliplatin	Oxaliplatin + DMB^®^
IC_50_	57	13	14	25	6.2	4.4	8.6	11	15	2
logIC_50_	1.75	1.11	1.13	1.39	0.79	0.64	0.93	1.02	1.16	0.22

## Data Availability

Research data supporting the results, analyzed, or generated during the study are available upon direct request from the authors and upon signing an agreement with the institution.

## Supplementary Materials

The following supporting information can be downloaded at: https://www.mdpi.com/article/10.3390/antiox14040381/s1, Table S1. Specifications of the compounds used for phenol extraction; Table S2. Specifications of the compounds used for quantification of total phenols; Table S3. Specifications of the compounds used for antioxidant activity assays; Table S4. Specifications of the compounds used for HPLC analysis (including CAS numbers, suppliers and 38 purity).
