# Supplementary material for: Anticancer Potential, Phenolic Profile, and Antioxidant Properties of Synsepalum dulcificum (Miracle Berry) in Colorectal Tumor Cell Lines
Source: Antioxidants (Basel). 2025 Mar 24;14(4):381. doi: 10.3390/antiox14040381 (PMC12024304; doi:10.3390/antiox14040381)
Supplement: Supplementary file 1 [file antioxidants-14-00381-s001.zip › antioxidants-3328968-supplementary.pdf]

Supplementary Materials

Table S1. Specifications of the compounds used for phenol extraction.

Compound	CAS number	Supplier	Purity
Ethanol	ETHA-9TA-1K0	Labkem	≥ 99.8 %

Table S2. Specifications of the compounds used for quantification of total phenols.

Compound	CAS number	Supplier	Purity
Folin-Ciocalteu's Reagent	A5084	PanReac AppliChem	≥ 99 %
Sodium carbonate	SOCA-A0A-500	Labkem	≥ 99.5 %
Galic acid	149-91-7	Sigma-Aldrich	≥ 98.0 %

Table S3. Specifications of the compounds used for antioxidant activity assays.

Compound	CAS number	Supplier	Purity
DPPH (2,2-difenil-1-picrylhydrazyl)	1898-66-4	Sigma Aldrich	≤ 100 %
ABTS (2,2′-Azino-bis(3-ethylbenzothiazoline-6-sulfonic acid) diammonium salt)	30931-67-0	Sigma Aldrich	≥ 98 %
Ammonium persulfate	7727-54-0	Sigma Aldrich	≥ 98 %
Trolox	AC218940050	Acros Organics	≥ 97 %

Table S4. Specifications of the compounds used for HPLC analysis (including CAS numbers, suppliers and purity).

Compound	CAS number	Supplier	Purity
Gallic acid	149-91-7	Sigma-Aldrich	≥ 97 wt. %
Protocatechuic Acid	99-50-3	Sigma-Aldrich	≥ 97 wt. %
Catechin	225937-10-0	Sigma-Aldrich	≥ 98 wt. %
Caffeic acid	331-39-5	Sigma-Aldrich	≥ 98 wt. %
Vanillin	121-33-5	Sigma-Aldrich	≥ 99 wt. %

Syringic acid	501-98-4	Sigma-Aldrich	≥ 95 wt. %
Rutin	207671-50-9	Sigma-Aldrich	≥ 94 wt. %
Cumaric acid	501-98-4	Sigma-Aldrich	≥ 98 wt. %
Ferulic acid	1135-24-6	Sigma-Aldrich	99 wt. %
Salicylic acid	69-72-7	Sigma-Aldrich	> 99 wt. %
Quercetin	849061-97-8	Sigma-Aldrich	≥ 95 wt. %
Cinamic acid	140-10-3	Sigma-Aldrich	≥ 99 wt. %
Acetonitrile (ACN)	75-05-8	Honeywell	HPLC grade
Water	7732-18-5	Honeywell	HPLC grade
Glacial Acetic acid	64-19-7	Labkem	≥ 99 wt. %
